# A decade of difference: cross-continental patterns of functional performance in Brazil and Europe within the WHO ICOPE framework

**DOI:** 10.1371/journal.pone.0353750

**Published:** 2026-08-05

**Authors:** Vinicius Rosa Oliveira, João Antônio Rodrigues Praia Júnior, Ricardo Dantas Dematte, Carlos Alberto Sanches, Felipe Farah Pinheiro Rodrigues, Eduard Minobes-Molina, André Librantz, Luciana Maria Malosá Sampaio

**Affiliations:** 1 Research group on Methodology, Methods, Models and Outcomes of Health and Social Sciences (M_3_O), Faculty of Health Sciences and Welfare, Centre for Health and Social Care Research (CESS), University of Vic-Central University of Catalonia (UVic-UCC), Vic, Spain; 2 Institute for Research and Innovation in Life Sciences and Health in Central Catalonia (IRIS-CC), Vic, Spain; 3 Informatics and Knowledge Management Graduate Program, Universidade Nove de Julho, São Paulo, Brazil; 4 Master’s and Doctorate in Rehabilitation Sciences Program, Universidade Nove de Julho, São Paulo, Brazil; AIIMS Jodhpur: All India Institute of Medical Sciences - Jodhpur, INDIA

## Abstract

**Introduction:**

Global aging patterns are highly heterogeneous, especially in diverse socioeconomic contexts.

**Objective:**

We aimed to analyze the determinants of functional performance within the WHO’s Integrated Care for Older People (ICOPE) framework.

**Methods:**

Functional performance was evaluated using basic and instrumental activities of daily living (BADL/IADL) from ELSI-Brasil and SHARE cohorts. Sociodemographic characteristics and the clinical domains of intrinsic capacity (mobility, vitality, cognition, psychological, and sensory functions) were also assessed.

**Results:**

The final sample consisted of 4,825 participants from ELSI-Brasil and 25,978 from SHARE. Functional risks diverged significantly based on intrinsic capacity domains: i) mobility: handgrip strength was a stronger protective factor in Europe, while the walking speed was a unique BADL risk factor in Brazil (OR 1.16); ii) vitality: weight loss showed a significant BADL risk for Europeans (OR 1.18); iii) cognition: low self-perceived memory showed similar risk for IADL across both cohorts (SHARE OR 1.23 vs. ELSI-Brasil OR 1.22); iv) psychological: depression posed a higher independent risk in Europe (OR 1.67–1.71) than in Brazil (OR 1.26–1.53), while poor self-rated health reflected higher risk in the Brazilian context (BADL OR 2.72 vs. 2.07 in Europe); v) sensory function: hearing impairment was a stronger IADL risk in Brazil (OR 1.27), while vision was significant only in Europe (OR 1.32).

**Conclusions:**

Functional decline begins a decade earlier in Brazil than in Europe, reflecting socioeconomic disparities across intrinsic capacity domains. ICOPE strategies should be tailored to each societal context to promote equitable healthy aging.

## Introduction

As life expectancy increases across the globe, the priority for public health initiatives has shifted from merely extending lifespan to optimizing the quality of life [1,2]. Activities of daily living (ADL) are the most common tool to assess functional abilities, being stratified according to complexity level as basic, instrumental, and advanced [3]. The basic activities of daily living (BADL) encompass fundamental self-care tasks such as bathing, dressing, and eating. The instrumental activities of daily living (IADL) involve tasks necessary for community dwelling including managing finances, cooking, and using transportation. The advanced ADL represent the highest level of functioning and reflect the ability of an individual to fulfill self-actualization and social roles, including care taking, legal planning, or online shopping [3]. The maintenance of high ADL levels is closely linked to an older person’s intrinsic capacity [4].

In response to the global need for person-centered health strategies, the World Health Organization (WHO) developed the Integrated Care for Older People (ICOPE) framework [5]. The ICOPE strategy focuses on the early identification of declines in intrinsic capacity domains, while crucially maintaining the functional performance (BADL and IADL) through individualized and coordinated care [5]. Complementing this perspective, the Fundamental Cause Theory posits that socioeconomic position and social support serve as primary drivers of health disparities because individuals with greater access to “flexible resources”, such as knowledge, financial means, and social networks, are better positioned to avoid risks and mitigate the consequences of disease [6]. Consequently, the relationship between intrinsic capacity and functional outcomes is shaped by environmental and structural conditions, which vary distinctively across populations as a result of socioeconomic inequalities, access to healthcare, and disparities in community resources [7]. Altogether, understanding how functional status manifests in different geopolitical contexts permits adapting and implementing the ICOPE framework effectively and equitably [5,8].

Brazil and Europe represent compelling contexts for examining BADL and IADL limitations among older adults, underscoring the need for context-specific approaches to implementing the ICOPE framework in these two distinct regions. European countries, characterized by advanced population aging, generally have more established long-term care infrastructures and social protection systems that support functional performance in later life [9]. In contrast, Brazil is undergoing one of the fastest demographic transitions worldwide, marked by profound socioeconomic inequalities, heterogeneous regional health profiles, and an urgent demand for integrated geriatric care strategies capable of addressing these disparities [10]. This study aimed to analyze the independent associations of BADL and IADL limitations among older adults in Brazil and Europe within the WHO ICOPE framework.

## Materials and methods

### Study design

This is a cross-sectional study that follows the STROBE statement (STrengthening the Reporting of OBservational studies in Epidemiology) [11]. Secondary data from two harmonized population-based cohorts were used: the Brazilian Longitudinal Study of Aging (ELSI-Brasil) and the Survey of Health, Ageing and Retirement in Europe (SHARE) [12,13]. Data were accessed for research purposes on the 5^th^ March 2025. The authors had no access to information that could identify individual participants during or after data collection. Physical, mental, and functional health were assessed using directly comparable indicators to examine determinants of intrinsic capacity, according to ICOPE guidelines [5].

The Cross-Industry Standard Process for Data Mining (CRISP-DM) methodology was adopted, which organizes the process into six interrelated phases: Problem Understanding, Data Understanding, Data Preparation, Modeling, Evaluation, and Deployment [14]. The CRISP-DM phases were adapted to the research project’s context. Problem understanding began with a systematic research process focused on identifying functional decline predictors in aging populations, followed by rigorous data preparation and preprocessing of ELSI-Brasil and SHARE datasets. The data preparation process involved comprehensive quality control procedures including verification of dataset dimensions, systematic treatment of missing values, exclusion of inconsistent or invalid observations, and appropriate handling of outliers. Afterwards, model development and balancing were performed using logistic regression, followed by thorough model evaluation and validation, and detailed statistical analysis with interpretation of odds ratios and confidence intervals. The results generated in this analysis were discussed by comparing forest plots and concordance analysis. This dual approach provided a set of consistent transnational risk factors and identified contextual disparities, offering robust insights into universal and population-specific determinants of functional decline in older adults.

### ELSI -Brasil database

#### Data source and study population.

ELSI-Brasil includes a nationally representative cohort of community-dwelling older adults aged 50 years or over. Longitudinal data was collected through interviews and assessments on the social, economic, and health dimensions of aging. The key investigated domains include functional capacity, cognitive performance, mental well-being, healthcare utilization patterns, a record of diagnosed chronic conditions, alongside metrics on socioeconomic status and lifestyle factors [12].

#### Procedures.

We used data from ELSI-Brasil second wave (2019–2021), which contains a sample of approximately 10,000 participants. The following data were collected from ELSI-Brasil database: sociodemographic characteristics, medical history, functional capacity, and cognitive function scores. Full methodological details of the original ELSI-Brasil study are published elsewhere [12].

### SHARE database

#### Data source and study population.

SHARE constitutes a fundamental reference for aging research, providing harmonized, longitudinal data on community-dwelling older adults aged 50 and over across 28 European countries and Israel [13]. Longitudinal data was collected through computer-assisted personal interviews on health indicators, economic circumstances, and social support systems. The key investigated domains include physical and mental health status, cognitive function, employment and pension status, wealth and consumption, family and social networks, and healthcare utilization patterns.

#### Procedures.

We used data from Wave 9, which contains a sample of approximately 70,000 participants across all participating countries. The following data was used in the present study: sociodemographic characteristics, health conditions, functional limitations, cognitive test scores, and economic variables. Full methodological details of SHARE are published elsewhere [15].

### Ethical considerations

ELSI-Brasil second wave (2019–2021) was approved by the Research Ethics Committee of the Oswaldo Cruz Foundation, Minas Gerais, Brazil (protocol 34649814.3.0000.5091). SHARE wave 9 (2021–2022) was approved by the Ethics Council of the Max Planck Society, Munich, Germany.

### Outcome variables

Outcome variables of both cohorts are based on respondent self-report, derived from “yes/no” questions about having difficulty to carry out specific tasks related to BADL and IADL. The former encompasses six core tasks fundamental to independent self-care: dressing, bathing, eating, transferring from a bed, using the toilet, and ambulating. BADL variables were condensed into a single binary variable, which captures whether participants have at least one BADL difficulty (coded as “yes” if they have at least one difficulty, and “no” if they have none). The same condensation process was applied to the IADL variables, indicating whether a participant has at least one IADL difficulty, that could include preparing a hot meal, shopping for groceries, using the telephone, and others (Table 1).

**Table 1:** Outcome variables collected from ELSI-Brasil and SHARE cohorts.

BADL variables	
ELSI-Brasil	SHARE
Difficulty moving from one room to another on the same floor	Difficulty walking across a room
Difficulty getting dressed	Difficulty getting dressed, including putting on socks and shoes
Difficulty bathing or showering	Difficulty bathing or showering
Difficulty to eat	Difficulty eating (e.g., cutting food)
Difficulty lying down or getting up from bed	Difficulty getting in or out of bed
Difficulty using the toilet	Difficulty using the toilet, including getting up or down from it
**IADL variables**	
ELSI-Brasil	SHARE
Difficulty preparing a hot meal	Difficulty preparing a hot meal
Difficulty using any form of transportation as a passenger	Difficulty leaving home independently and using public transportation
Difficulty shopping for groceries	Difficulty shopping for groceries
Difficulty using the telephone	Difficulty using the telephone
Difficulty managing one’s own medications	Difficulty taking medicines
Difficulty managing one’s own money	Difficulty managing one’s own money
Difficulty performing personal hygiene/ grooming	
Difficulty performing light housework	
Difficulty performing heavy housework	

BADL: basic activities of daily living; IADL: instrumental activities of daily living.

### Selection of explanatory variables

The key intrinsic capacity domains outlined in the ICOPE framework were used to select explanatory variables [5]. For the mobility domain, assessment included walking speed (only available in ELSI-Brasil) and handgrip strength measurements. Vitality was assessed through self-reported unintentional weight loss. Cognition was assessed through self-reported memory, while psychological status was measured through self-rated health and depressive symptom inventories available in both databases. Sensory capacity was evaluated through self-reported visual acuity (both near and far vision, with corrective lenses if typically used) and self-reported hearing capacity (with hearing aids if normally used), according to current community-level assessment strategies [5]. In alignment with ICOPE’s comprehensive assessment approach, which combines both clinical evaluation and self-reported measures, we included incontinence and a self-rated health variable [5].

### Sociodemographic and clinical covariates

Sociodemographic covariates included sex, age, and educational attainment. Comorbidity burden was included as clinical covariates. To facilitate cross-continental comparison, consistent categorization was applied across databases. Age was discretized into four categories (‘50-59’, ‘60-69’, ‘70-79’, ‘80+’) following SHARE’s classification. Educational attainment was harmonized into a four-level ordinal variable (‘no formal education’, ‘elementary school’, ‘high school’, ‘higher education’ for ELSI-Brasil; ‘0-6 years’, ‘7-12 years’, ‘13-18 years’, ‘19+ years’ for SHARE [13].

### Variable standardization procedures

Handgrip strength measurements were standardized to account for age and sex-related physiological differences. For ELSI-Brasil, the mean of three measurements was calculated for each participant, and standard scores were computed based on age- and sex-specific distributions. A parallel standardization procedure was applied to SHARE’s four handgrip measurements (recorded on a 0–100 scale). The walking speed was measured in seconds, representing the time taken to walk a 3-meter distance at the participant’s own pace, and was standardized similarly.

### Harmonization of categorical variables

Categorical variables were harmonized to ensure comparability between studies. Incontinence was dichotomized from its original categories in ELSI-Brasil (no incontinence, urinary, fecal, or double incontinence) to align with SHARE’s binary assessment (use of incontinence pads: yes/no). Self-reported memory, general health, vision, and hearing variables — originally measured on five-point ordinal scales with differing descriptors — were recorded into consistent three-level categories (good, fair, poor). For vision and hearing, a composite variable was created reflecting the poorest level reported across specific visual or auditory functions.

The multimorbidity variable was defined as self-report of three or more chronic conditions across both cohorts. To ensure comparability, we standardized the conditions reported by ELSI-Brasil and SHARE. The common set of eleven conditions used for the pooled analysis was: hypertension, diabetes, high cholesterol, heart diseases, stroke, pulmonary diseases, rheumatoid arthritis, osteoporosis, cancer, kidney failure, Parkinson’s disease, Alzheimer’s disease, and cataract. While the ELSI-Brasil survey included a greater specificity for certain heart events and procedures (including heart attack, angina, and heart failure), the final variable was mapped to the broader SHARE categories to maintain comparability across the analysis [16,17].

The depression variable consisted of depressive symptoms assessed through scales in both ELSI-Brasil and SHARE, combined with the self-reported depression variable from each cohort. Depression was defined as having four or more symptoms or a self-reported diagnosis [16].

### Statistical analysis

The analysis was performed using Python version 3.13.2 (Python Software Foundation, Beaverton, OR). A multivariate binomial logistic regression was employed to identify factors associated with the outcome variables. In the multicollinearity analysis, all variables presented a variance inflation factor below 5, indicating no significant collinearity. Given the anticipated age differences between the two population-based cohorts, age was included as a covariate in all regression models to control for its potential confounding effect on the functional outcomes under study. Model fit was evaluated using the Hosmer-Lemeshow test. The significance of the associations was determined based on the odds ratio (OR), its 95% confidence interval (95% CI), and a *p*-value of < 0.05.

## Results

### Sociodemographics

**Fig 1:**
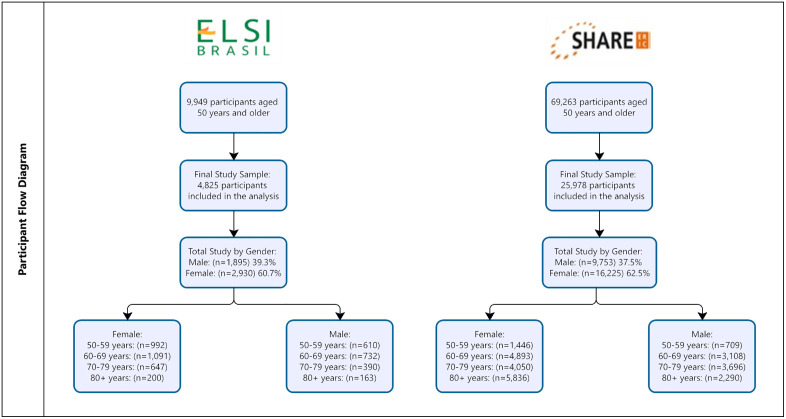
Flow diagram of the studied population.

Following rigorous data processing, final analytical samples of 4,825 participants from ELSI-Brasil and 25,978 from SHARE were established (Fig 1). Participant sociodemographic and clinical characteristics are summarized in Table 2. The full set of variables, stratified by age group and sex, is available in S1 and S2 Tables.

**Table 2:** Demographic and clinical characteristics of the sample.

Variables	ELSI-Brasil	SHARE
**Anthropometric**	**Mean (SD)**	**Mean (SD)**
Age (years)	64.9 (9.0)	72.6 (9.4) †
Weigth (kg)	71.2 (14.9)	78.5 (16.0)
Height (m)	1.59 (0.09)	1.67 (0.09)
BMI (kg/m²)	28.0 (5.1)	27.9 (5.1)
**Gender**	**n (%)**	**n (%)**
Female	2,930 (60.7)	16,225 (62.5)
Male	1,895 (39.3)	9,753 (37.5)
**Schooling**	**n (%)**	**n (%)**
Never studied (ELSI-Brasil) / 0–6 years (SHARE)	577 (12.0)	3,320 (12.8)
Elementary school (ELSI-Brasil) / 7–12 years (SHARE)	2,846 (59.0)	14,235 (54.8)
High school (ELSI-Brasil) / 13–18 years (SHARE)	998 (20.7)	7,594 (29.2)
Higher education (ELSI-Brasil) / 19 + years (SHARE)	404 (8.4)	829 (3.2)

† p < 0.001, calculated using Welch’s independent samples t-test

BMI: body mass index; kg/m²: kilogram per square meter; SD: standard deviation

### BADL

**Fig 2:**
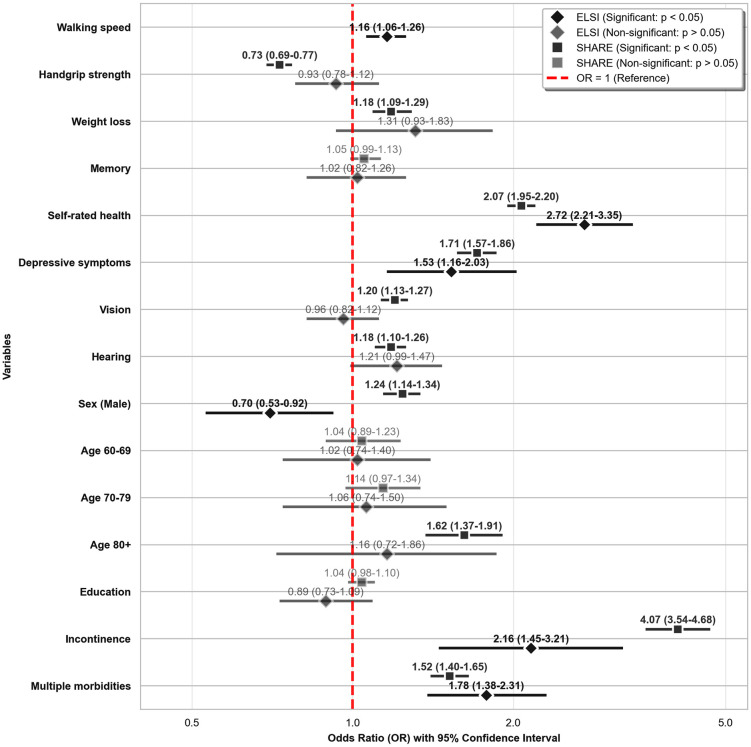
Associations for basic activities of daily living (BADL) in ELSI-Brasil and SHARE cohorts. Forest plot displays the Odds Ratios (OR) and 95% Confidence Intervals (CI) derived from multivariable logistic regression models for both studies, enabling a visual assessment of the differences in association strengths. An Odds Ratio (OR) > 1 indicates a risk factor for limitations in BADL, while an OR < 1 suggests a protective factor. A p-value below 0.05 was considered statistically significant.

Factors associated with impairment in at least one BADL showed pronounced differences between the Brazilian and European cohorts (Fig 2). Male sex was as a protective factor against BADL impairment in Brazil (OR=0.70) but acted as a risk factor in Europe (OR=1.24). Functional performance risk was notably concentrated in the oldest age group in Europe, where only individuals aged 80 years or older showed a significant risk (OR=1.62).

Clinical factors varied significantly across the populations. Incontinence posed a near two-fold higher risk for Europeans (OR=4.07) compared to Brazilians (OR=2.16). Conversely, the risk associated with multiple comorbidities was higher in Brazil (OR=1.78) than in Europe (OR=1.52). While low self-rated health was a significant risk factor in both cohorts, its effect was stronger for Brazilians (OR=2.72) than for Europeans (OR=2.07). Low handgrip strength acted as a protective factor only for the European population (OR=0.73), showing no significant association for Brazilians.

Sensory deficits and vitality were key differentiating factors. Hearing, vision, and weight loss were all statistically insignificant risk factors for BADL ability in the Brazilian cohort. However, all three factors emerged as significant risk factors for Europeans (OR=1.18, OR=1.20, and OR=1.18, respectively). Furthermore, depressive symptoms were a strong risk factor for BADL ability in both groups, with stronger association in Europe (OR=1.71) compared to Brazil (OR=1.53). A direct measure of physical performance, the walking speed, was exclusive to the ELSI-Brazil cohort and confirmed a risk association with BADL ability (OR=1.16).

### IADL

**Fig 3:**
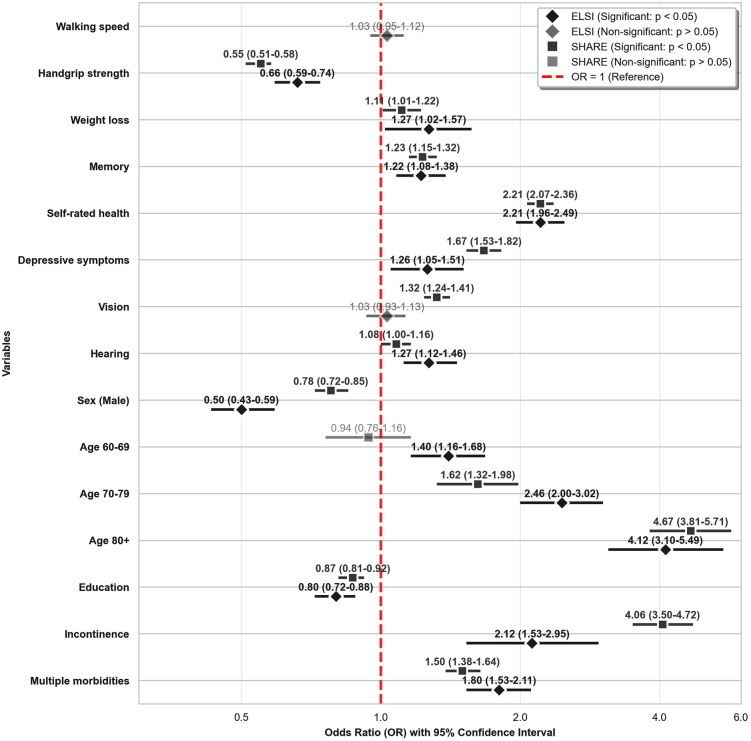
Associations for instrumental activities of daily living (IADL) in ELSI-Brasil and SHARE cohorts. Forest plot displays the Odds Ratios (OR) and 95% Confidence Intervals (CI) derived from multivariable logistic regression models for both studies, enabling a visual assessment of the differences in association strengths. An Odds Ratio (OR) > 1 indicates a risk factor for limitations in IADL, while an OR < 1 suggests a protective factor. A p-value below 0.05 was considered statistically significant.

Factors associated with impairment in at least one IADL in Brazilians and Europeans are presented in Fig 3. Key sociodemographic factors consistently acted as protective elements across both cohorts. Male sex was a protective factor in both cohorts, with a notably stronger effect observed among Brazilians (OR=0.50) than Europeans (OR=0.78). Higher education levels were also protective against IADL impairment, showing a slightly stronger effect in Brazil (OR=0.80) than in Europe (OR=0.87). Functional dependence on IADL emerged earlier in the Brazilian population, starting at 60–69 years (OR=1.40), whereas the risk significantly began later for Europeans, starting at 70–79 years (OR=1.62). The risk then increased gradually in both cohorts in subsequent age groups.

Analysis of clinical and functional factors revealed notable geographical and population-specific differences in the risk of IADL impairment. Incontinence emerged as a risk factor in both cohorts, but its effect was nearly twice as strong in Europeans (OR=4.06) compared to Brazilians (OR=2.12). Similarly, multiple comorbidities increased the risk in both populations, yet the effect was higher among Brazilians (OR=1.80) than Europeans (OR=1.50). Conversely, low self-rated health demonstrated a statistically identical risk effect across both cohorts (ELSI-Brasil OR=2.21; SHARE OR=2.21). Handgrip strength was consistently protective and showed a slight stronger effect in Europe (OR=0.55) than in Brazil (OR=0.66).

The comparative analysis of sensory and cognitive determinants of IADL ability revealed several key distinctions between the populations. Depressive symptoms significantly increased the risk of impairment in both cohorts, although the effect was noticeably stronger for Europeans (SHARE OR=1.67) than for Brazilians (ELSI-Brasil OR=1.26). Similarly, low self-perceived memory functioned as a consistent risk factor in both groups (Europe OR=1.23; Brazil OR=1.22). However, specific sensory factors showed differing transnational effects: hearing impairment was a more pronounced risk factor among Brazilians (OR=1.27) compared to Europeans (OR=1.08), while vision impairment was found to be a statistically significant risk factor only in the European population (OR=1.32).

## Discussion

This population-based study examined the independent associations between measures of intrinsic capacity domains and functional performance (BADL and IADL) in Brazilian and European settings. We identified significantly diverse functional performance risk profiles between the two cohorts. The core distinction lies in the structural environment, where European countries typically reflect established welfare states aiming for lowering morbidity levels, whereas Brazil demonstrates an aging trajectory where functional limitations are experienced earlier and amplified by systemic socioeconomic inequality.

Functional dependence on IADL emerges earlier in Brazil, beginning at the age 60–69 years, whereas the significant risk onset in Europe occurs a decade later. This accelerated aging trajectory goes in line with the Theory of Cumulative Disadvantage [18]. Functional limitations are heavily concentrated among those with lower education and income because of lifelong exposures to poverty and lower educational attainment [19]. These factors accelerate the onset of chronic diseases and subsequent functional loss, requiring formalized support structures or informal care a full decade earlier than observed in high-income countries.

Higher education levels consistently protect against IADL impairment, showing a slightly stronger effect in Brazil than in Europe. A previous analysis of ELSI-Brasil (Wave 1, 2015–2016) revealed primary school as the most prevalent education level, equivalent to 1–4 years of education in the age group 50–59 years [18]. This suggests that lower education is related to inferior access to healthcare, hindering older adults to effectively prolong their functional independence.

The protective effect by male sex observed for IADL can be considered a product of cultural IADL measurement bias. Dependent older men in Brazil often benefit from strong informal care networks that provide extensive personal support, buffering the impact of functional loss on measured independence [20]. In contrast, the finding that male sex is a risk factor for BADL in Europe aligns with the male morbidity paradox observed in high-income countries, where men often maintain independence longer but suffer a steeper, more catastrophic functional decline when it occurs [21]. Nevertheless, gender comparisons should attend to the gender-specific measurement properties of the items which BADL or IADL are comprised for more accurate interpretation [22].

According to our findings, incontinence was associated with twice the risk for BADL and IADL limitations in Europe compared to Brazil. This counterintuitive finding can be explained by cultural stigma and measurement distortion because incontinence is heavily stigmatized and often perceived as an inherent, unchangeable part of aging, leading to reluctance to discuss the issue with healthcare providers in many developing countries [23]. Therefore, we postulate the strong cultural taboo plays a role in incontinence underreporting in the Brazilian community-dwelling sample.

The presence of multiple comorbidities in Brazil translates into functional limitation, and is accelerated by factors such as low socioeconomic status, inadequate nutrition, and suboptimal chronic disease management. On the one hand, individuals with high socioeconomic status have greater access to diagnosis, resulting in a reported morbidity that is strongly associated with healthcare access [24]. On the other hand, people with chronic conditions (whether diagnosed or not) reaches an advanced stage of severity, where clinical pathology inevitably dictates functional outcome [25,26].

We explored the associations of intrinsic capacity with functional limitations in BADL and IADL cross-continentally. In the mobility domain, the slight lower protection of handgrip strength in Brazil could suggest the influence of ethnic differences in skeletal muscle mass and socioeconomic status. Other authors have explored differences in handgrip strength across low- and middle-income countries, showing a strong relationship between wealth and handgrip strength [27,28]. The significance of the walking speed in Brazil suggests that dynamic, performance-based measures of locomotion, which integrate multiple aspects of the musculoskeletal and neurological systems, are more robust and sensitive predictors of functional decline than static strength measurements in populations with diverse occupational histories [29].

The combination of sensory and vitality domains of intrinsic capacity underpins structural healthcare deficits across the analyzed cohorts. We identified hearing, vision, and weight loss as significant risk factors for BADL ability in Europe. A systematic review supports the potential association between the presence of single or multiple sensory impairments and a greater likelihood of sarcopenia, which could explain the functional limitations observed [30]. Additionally, recent literature suggests the key role of early screening of sensory impairments as insidious drivers of cognitive and physical decline in high-income countries [31]. The lack of significance in the Brazilian context could imply failure in screening these conditions consistently in the population.

The nearly identical risk associated with low self-perceived memory across both populations points cognitive capacity as a universal driver of functional decline. This effect suggests that the subjective cognitive struggle by an individual and the subsequent inability to manage complex tasks is fundamentally hardwired into the aging process, regardless of the person’s geographic location or wealth. Our findings are supported by a meta-analysis that showed significant association of IADL with verbal and spatial memory, thereby confirming association of global cognitive functioning with functional performance [32].

We identified stronger association between depressive symptoms and functional impairment in the European population. High-income countries, in general, have higher prevalence of major depressive disorders compared to low- and middle-income countries [33]. Methodological issues could influence these estimates, such as differences in symptom expression across cultures, which are modulated by constructs including loneliness, social expectations and acceptability of communicating affective states [33].

Self-rated health is a robust subjective measure reflecting overall physical and mental state. According to ELSI-Brasil Wave 1 (2015–2016), 11.5% of older adults rated their health as poor and very poor [18]. The most contributing factors were income, education and having a private health insurance plan [18]. Therefore, when Brazilian older adults report poor self-rated health and exhibit BADL impairment, it often signals a catastrophic confluence of multimorbidity and lack of adequate care.

The findings of this study highlight important clinical and policy implications for promoting healthy aging within the WHO ICOPE framework. From a clinical perspective, they support the need for integrated, multidisciplinary care models that incorporate early screening of functional, sensory, and psychological domains, along with targeted interventions such as strength training and mobility programs to preserve intrinsic capacity. From a policy standpoint, the observed cross-national disparities underscore the importance of adapting ICOPE strategies to diverse socioeconomic contexts, including: (i) the implementation of low-cost screening for modifiable risk factors (e.g., vision, hearing, and nutritional status); (ii) the cultural validation of functional assessment tools to address gender-related reporting biases; and (iii) the development of policies that reduce structural inequalities by enhancing education, financial security, and equitable access to chronic disease care, ultimately supporting longer and more independent aging trajectories.

This study presents some limitations. Firstly, the reliance on cross-sectional data prevents the establishment of definitive causality, merely reporting associations through odds ratios. Secondly, several variables were derived from self-reported measures, which may introduce bias. For instance, multimorbidity was based on self-reported physician diagnoses, potentially overrepresenting individuals with greater access to healthcare services. Similarly, sensory function (hearing and vision) and functional capacity (BADL and IADL) were assessed through self-report, making them susceptible to misclassification and reporting bias. Nevertheless, older adults generally identify meaningful sensory and functional limitations, particularly when these interfere with daily activities. We also acknowledge the possibility of differential reporting by sex, as men may underreport functional limitations due to sociocultural perceptions of dependency and masculinity. However, given the large and diverse samples analyzed, such bias is likely to be non-differential and unlikely to fully account for the observed associations. Future studies integrating self-reported and objective measures are warranted to strengthen the robustness of these findings. Finally, data collection for both ELSI-Brasil (Wave 2) and SHARE (Wave 9) occurred during the COVID-19 pandemic, a context in which social isolation and reduced physical activity have been associated with declines in physical and cognitive function among older adults [34,35]. In Brazil, data were collected during more severe phases of the pandemic, whereas in Europe they corresponded to a period of gradual normalization. These differences may have introduced contextual variability between cohorts, potentially influencing some of the dimensions assessed in this study (e.g., functional status, mobility, depressive symptoms, and healthcare utilization).

The main strength of this study is the robust and rigorous analysis of two large cohort samples representing a rapidly aging upper middle-income context (ELSI-Brasil) and established high-income European welfare states (SHARE). This dual-cohort approach yields policy-relevant data crucial for tailoring integrated care strategies to address global aging disparities.

## Conclusions

Functional decline begins a decade earlier in Brazil than in Europe, showing different risk profiles across intrinsic capacity domains. This reflects that the determinants of functional performance are shaped by socioeconomic structure and healthcare context. Effective ICOPE strategies should acknowledge these cross-continental differences, demanding early, targeted interventions that address structural inequality to truly compress morbidity in middle-income settings.

## Supporting information

S1 Table Demographic and clinical variables stratified by age group and sex in ELSI-Brasil cohort. (DOC)

S2 Table Demographic and clinical variables stratified by age group and sex in SHARE cohort. (DOC)
