# Supplementary material for: A decade of difference: cross-continental patterns of functional performance in Brazil and Europe within the WHO ICOPE framework
Source: PLoS One. 2026 Aug 5;21(8):e0353750. doi: 10.1371/journal.pone.0353750 (PMC13440855; doi:10.1371/journal.pone.0353750)
Supplement: Supplementary file 1 [file pone.0353750.s004.docx]

|  | 2^nd^ Jun 2026 |
| --- | --- |

Dear Dr. Yogesh Kumar Jain,

We are pleased to enclose, for your consideration, the responses to each point raised by the academic editor and reviewer(s) to the manuscript **PONE-D-26-11227** "A decade of difference: cross-continental patterns of functional performance in Brazil and Europe within the WHO ICOPE framework”.

Additional Editor Comments:
The manuscript has received an overall positive feedback by the reviewers. However, I would recommend some minor revisions based on the suggestions, especially from reviewer #2. This would greatly improve the clarity of the messages conveyed.

**R: Thank you for your positive feedback. The comments of both reviewers have been addressed accordingly.**

Review Comments to the Author

Reviewer #1: The manuscript presents clear and coherent objectives, and the methodology is appropriate to address the research question, with a sufficient sample size and the use of valid and reliable instruments. The results are clearly presented, and the conclusions are supported by the data. The statistical approach appears adequate for the aims of the study. In addition, the study uses data derived from two public datasets/projects, and therefore the underlying data are publicly available, supporting transparency and reproducibility. The manuscript is well written, clear, and easy to understand. The objectives, methods, and results are presented in a logical and coherent manner, and the English language is appropriate for scientific publication.

**R: We are pleased that the reviewer found the manuscript to be well written, logically structured, and easy to follow. We have carefully revised the manuscript to ensure that this level of clarity is maintained throughout and have made minor editorial improvements where appropriate. Thank you for your valuable feedback and support.**

Reviewer #2: I congratulate the authors on the manuscript.
Some issues could be better described: was the self-reported sensory assessment (vision and hearing) based on any literature? If so, they should cite it.

**R: Thank you for this comment. Actually, the self-reported sensory assessment (vision and hearing) used both in ELSI-Brasil and SHARE are subjectivelly collected using a 5-point Likert-type scale. These assessments in both datasets have common similarities such as the inclusion of correction devices (glasses/hearing aids) in the evaluation, and the use of ordinal scales reflecting perceived ability. Overall, they capture functional sensory capacity rather than clinical diagnosis, in accordance with the WHO ICOPE framework. The references have been cited accordingly (line 150).**

Do the authors believe that elderly people know how to identify hearing loss?

**R: Thank you for this important and thought-provoking comment. While self-reported hearing measures rely on an individual’s perception, evidence suggests that older adults are able to reasonably identify functional hearing difficulties, particularly when these limitations affect daily communication. Given that both ELSI-Brasil and SHARE use harmonized self-reported questions (including the use of hearing aids), these measures remain appropriate and widely accepted for large population-based studies, particularly in cross-national comparisons where objective testing is not feasible. We have added a brief clarification in the manuscript acknowledging this limitation (lines 350-353).**

Table 2 shows that the average ages of the two groups appear to be quite different. Was a statistical analysis performed on this? Could this have influenced the results?

**R: We thank the reviewer for this important observation. We performed Welch's independent samples t-test to formally compare the mean age between the two cohorts, which confirms the statistically significant difference (ELSI-Brasil: 64.9 ±
9.0 years; SHARE: 72.6 ± 9.4 years; t = -54.19, df = 6924.03, p < 0.001).**

**We fully agree that age is a critical factor that could potentially influence the results, given its strong association with funcional decline. Therefore, age was pre-defined and included as a sociodemographic covariate in all multivariable logistic regression
models. The reported associations for all outcomes are thus statistically adjusted for age, sex, and education, which substantially minimizes the risk of confounding by the age difference between the two samples. We have now added a clarifying statement in the Methods section explaining the inclusion of age as a covariate to control for potential confounding (lines 190-192), and we have inserted the p-value in Table 2 (footnote, line 203).**

Of course we have to believe the participants' answers, but my experience when evaluating IADLs and BADLs is that men lie in their responses. Do you have anything to say about that?

**R:** **Thank you for raising this important point. We agree that self-reported functional assessments may be influenced by gender-related social expectations and reporting behavior, particularly among men, who may underreport dependency due to norms associated with masculinity and autonomy. Accordingly, we acknowledge that self-reported measures of BADLs and IADLs may be subject to reporting bias, including potential differences by sex. However, self-reported functional assessments remain the standard approach in large population-based studies such as ELSI-Brasil and SHARE, given their feasibility and their ability to capture perceived functional capacity in daily life, thereby ensuring consistency and comparability across populations. We have acknowledged this as a potential limitation in the manuscript and highlighted the need for future studies combining self-reported and objective functional measures (lines 353–358).**

Typically, handgrip strength assessment is used to evaluate the vitality domain. Why did the authors use it in the locomotor domain?

**R: Thank you for this insightful comment. In our study, handgrip strength was included within the mobility domain because it reflects neuromuscular performance and functional reserve associated with locomotion and disability risk. Although measured in the upper limb, handgrip strength is widely recognized as a proxy for global muscle strength, and has been strongly associated with mobility outcomes such as gait speed, chair rise performance, and risk of physical disability and frailty (Abdalla et al., 2023, doi:** [**10.1016/j.archger.2022.104869**](https://pubmed.ncbi.nlm.nih.gov/36442405/)**; McGrath et al., 2022, doi:** [**10.1016/j.archger.2022.104716**](https://pubmed.ncbi.nlm.nih.gov/35569287/)**). This approach also ensured consistency across both datasets, where comparable locomotor performance measures were limited.**

The data collection for both ELSI-Brazil (second wave) and SHARE (ninth wave) occurred during the COVID-19 pandemic. Could this have interfered? The ELSI-Brazil study was collected during the worst phase of the pandemic, while SHARE was collected when the pandemic was more controlled in Europe. Could this have interfered? None of this was discussed in the manuscript.

**R: We thank the reviewer for raising this highly relevant point. We agree that ELSI-Brasil Wave 2 (2019-2021) was collected during a more critical phase of the COVID-19 pandemic in Brazil, whereas SHARE Wave 9 (2021-2022) occurred during a comparatively more controlled phase in Europe. Therefore, the pandemic context may have influenced functional performance, mental health, mobility, and self-perception outcomes in both cohorts. However, pandemic-related factors are unlikely to fully account for the observed patterns, especially given the consistency of key associations across domains and populations. We added this limitation into the Discussion (lines 358-364) and thank the reviewer for helping us improve the contextual interpretation of our findings.**

I missed seeing practical and clinical applications for the results. What suggestions do the authors have for improving healthy aging, considering the study's findings?

**R:** **We thank the reviewer for prompting us to strengthen the clinical relevance of our work. We have now incorporated the clinical implications of our findings alongside the policy implications in the Discussion section (lines 335-345).**

The authors should state the limitations of the study.

**R: The “limitations” paragraph has been updated in accordance with the reviewer’s comments (lines 346-364).**

We sincerely appreciate the reviewer’s careful evaluation and believe the manuscript has been substantially strengthened following these recommendations.

Yours sincerely,

The authors
