# Supplementary material for: A decade of difference: cross-continental patterns of functional performance in Brazil and Europe within the WHO ICOPE framework
Source: PLoS One. 2026 Aug 5;21(8):e0353750. doi: 10.1371/journal.pone.0353750 (PMC13440855; doi:10.1371/journal.pone.0353750)
Supplement: Supplementary file 2 [file pone.0353750.s001.doc]

**S1Table.** **Demographic and clinical variables stratified by age group and sex in ELSI-Brasil cohort.**

| **ELSI-Brasil cohort** | | | | | | | | | | |
| --- | --- | --- | --- | --- | --- | --- | --- | --- | --- | --- |
| **Variable** | **Category** | **All (n=4,825)** | **Female 50-59 years (n=992)** | **Male 50-59 years (n=610)** | **Female 60-69 years (n=1,091)** | **Male 60-69 years (n=732)** | **Female 70-79 years (n=647)** | **Male 70-79 years (n=390)** | **Female 80+ years (n=200)** | **Male 80+ years (n=163)** |
| **Weight (kg)** |  | 4,825 (100.0%) | 992 (61.9%) | 610 (38.1%) | 1,091 (59.8%) | 732 (40.2%) | 647 (62.4%) | 390 (37.6%) | 208 (57.3%) | 155 (42.7%) |
|  | Mean | 71.2 | 70.4 | 78,8 | 69.3 | 76.6 | 65.4 | 72.5 | 61.6 | 67.6 |
|  | SD | 14.9 | 13.8 | 15.7 | 14.4 | 14.6 | 13.4 | 12.6 | 12.7 | 13.5 |
| **Height (m)** |  | 4,825 (100.0%) | 992 (61.9%) | 610 (38.1%) | 1,091 (59.8%) | 732 (40.2%) | 647 (62.4%) | 390 (37.6%) | 208 (57.3%) | 155 (42.7%) |
|  | Mean | 1.59 | 1.56 | 1.68 | 1.52 | 1.66 | 1.52 | 1.65 | 1.51 | 1.63 |
|  | SD | 0.09 | 0.07 | 0.07 | 0.07 | 0.07 | 0.06 | 0.07 | 0.08 | 0.08 |
| **BMI (kg/m²)** |  | 4,825 (100.0%) | 992 (61.9%) | 610 (38.1%) | 1,091 (59.8%) | 732 (40.2%) | 647 (62.4%) | 390 (37.6%) | 208 (57.3%) | 155 (42.7%) |
|  | Mean | 28.0 | 28.7 | 27,6 | 28.8 | 27.6 | 28.0 | 26.5 | 26.9 | 25.3 |
|  | SD | 5.1 | 5.2 | 4.48 | 5.48 | 4.62 | 5.32 | 4.14 | 4.87 | 4.06 |
| **Years of schooling** | Never studied | 577 (12.0%) | 76 (7.7%) | 22 (3.6%) | 111 (10.2%) | 63 (8.6%) | 126 (19.5%) | 70 (17.9%) | 69 (33.2%) | 40 (25.8%) |
|  | Elementary school | 2,846 (59.0%) | 523 (52.7%) | 343 (56.2%) | 658 (60.3%) | 458 (62.6%) | 399 (61.7%) | 255 (65.4%) | 115 (55.3%) | 95 (61.3%) |
|  | High school | 998 (20.7%) | 282 (28.4%) | 186 (30.5%) | 223 (20.4%) | 155 (21.2%) | 84 (13.0%) | 46 (11.8%) | 10 (4.8%) | 12 (7.7%) |
|  | Higher education | 404 (8.4%) | 111 (11.2%) | 59 (9.7%) | 99 (9.1%) | 56 (7.7%) | 38 (5.9%) | 19 (4.9%) | 14 (6.7%) | 8 (5.2%) |
| **Incontinence** |  | 231 (4.8%) | 35 (3.5%) | 17 (2.8%) | 48 (4.4%) | 17 (2.3%) | 46 (7.1%) | 22 (5.6%) | 26 (12.5%) | 20 (12.9%) |
| **Walking speed** |  | 1,598 (33.5%) | 610 (38.1%) | 992 (61.9%) | 732 (40.2%) | 1091 (59.8%) | 390 (37.6%) | 647 (62.4%) | 155 (42.7%) | 208 (57.3%) |
| **Handgrip strength** |  | 1,602 (33.0%) | 992 (61.9%) | 610 (38.1%) | 1,091 (59.8%) | 732 (40.2%) | 647 (62.4%) | 390 (37.6%) | 208 (57.3%) | 155 (42.7%) |
| **Weight loss** |  | 526 (10.9%) | 106 (10.7%) | 62 (10.2%) | 148 (13.6%) | 58 (7.9%) | 77 (11.9%) | 38 (9.7%) | 21 (10.1%) | 16 (10.3%) |
| **Multiple morbidities (≥3)** |  | 1,319 (27.3%) | 185 (18.6%) | 73 (12.0%) | 362 (33.2%) | 145 (19.8%) | 288 (44.5%) | 102 (26.2%) | 103 (49.5%) | 61 (39.4%) |
| **Depressive symptoms** |  | 896 (18.6%) | 268 (27.0%) | 89 (14.6%) | 262 (24.0%) | 73 (10.0%) | 118 (18.2%) | 36 (9.2%) | 32 (15.4%) | 18 (11.6%) |
| **Self-rated health** | Good | 2,442 (50.6%) | 506 (51.0%) | 345 (56.6%) | 547 (50.1%) | 380 (51.9%) | 319 (49.3%) | 188 (48.2%) | 89 (42.8%) | 68 (43.9%) |
|  | Regular | 1,846 (38.3%) | 380 (38.3%) | 205 (33.6%) | 428 (39.2%) | 279 (38.1%) | 245 (37.9%) | 161 (41.3%) | 88 (42.3%) | 60 (38.7%) |
|  | Poor | 537 (11.1%) | 106 (10.7%) | 60 (9.8%) | 116 (10.6%) | 73 (10.0%) | 83 (12.8%) | 41 (10.5%) | 31 (14.9%) | 27 (17.4%) |
| **Memory** | Good | 531 (11.0%) | 94 (9.5%) | 97 (15.9%) | 113 (10.4%) | 85 (11.6%) | 64 (9.9%) | 43 (11.0%) | 16 (7.7%) | 19 (12.3%) |
|  | Regular | 2,377 (49.3%) | 488 (49.2%) | 308 (50.5%) | 522 (47.8%) | 380 (51.9%) | 316 (48.8%) | 201 (51.5%) | 98 (47.1%) | 64 (41.3%) |
|  | Poor | 1,917 (39.7%) | 410 (41.3%) | 205 (33.6%) | 456 (41.8%) | 267 (36.5%) | 267 (41.3%) | 146 (37.4%) | 94 (45.2%) | 72 (46.5%) |
| **Vision** | Good | 2,646 (54.8%) | 522 (52.6%) | 333 (54.6%) | 617 (56.6%) | 399 (54.5%) | 370 (57.2%) | 221 (56.7%) | 105 (50.5%) | 79 (51.0%) |
|  | Regular | 1,284 (26.6%) | 270 (27.2%) | 172 (28.2%) | 270 (24.7%) | 205 (28.0%) | 152 (23.5%) | 105 (26.9%) | 68 (32.7%) | 42 (27.1%) |
|  | Poor | 895 (18.6%) | 200 (20.2%) | 105 (17.2%) | 204 (18.7%) | 128 (17.5%) | 125 (19.3%) | 64 (16.4%) | 35 (16.8%) | 34 (21.9%) |
| **Hearing** | Good | 3,661 (75.9%) | 795 (80.1%) | 478 (78.4%) | 876 (80.3%) | 536 (73.2%) | 470 (72.6%) | 274 (70.3%) | 140 (67.3%) | 92 (59.4%) |
|  | Regular | 948 (19.6%) | 169 (17.0%) | 118 (19.3%) | 169 (15.5%) | 158 (21.6%) | 143 (22.1%) | 92 (23.6%) | 51 (24.5%) | 48 (31.0%) |
|  | Poor | 216 (4.5%) | 28 (2.8%) | 14 (2.3%) | 46 (4.2%) | 38 (5.2%) | 34 (5.3%) | 24 (6.2%) | 17 (8.2%) | 15 (9.7%) |
| **BADL limitation (>1)** |  | 300 (6.2%) | 59 (5.9%) | 28 (4.6%) | 76 (7.0%) | 31 (4.2%) | 56 (8.7%) | 18 (4.6%) | 24 (11.5%) | 8 (5.2%) |
| **IADL limitation (>1)** |  | 1,372 (28.4%) | 213 (21.5%) | 96 (15.7%) | 347 (31.8%) | 120 (16.4%) | 298 (46.1%) | 105 (26.9%) | 126 (60.6%) | 67 (43.2%) |

BADL: basic activities of daily living; BMI: body mass index; IADL: instrumental activities of daily living; kg/m²: kilogram per square meter; SD: standard deviation.
