# Supplementary material for: A decade of difference: cross-continental patterns of functional performance in Brazil and Europe within the WHO ICOPE framework
Source: PLoS One. 2026 Aug 5;21(8):e0353750. doi: 10.1371/journal.pone.0353750 (PMC13440855; doi:10.1371/journal.pone.0353750)
Supplement: Supplementary file 3 [file pone.0353750.s002.doc]

**S2 Table.** **Demographic and clinical variables stratified by age group and sex in SHARE cohort.**

| **SHARE cohort** | | | | | | | | | | |
| --- | --- | --- | --- | --- | --- | --- | --- | --- | --- | --- |
| **Variables** | **Category** | **All (n=25,978)** | **Female 50-59 years (n=1,446)** | **Male 50-59 years (n=709)** | **Female 60-69 years (n=4,893)** | **Male 60-69 years (n=3,108)** | **Female 70-79 years (n=4,050)** | **Male 70-79 years (n=3,696)** | **Female 80+ years (n=5,836)** | **Male 80+ years (n=2,290)** |
| **Weight (kg)** |  | 25,978 (100%) | 1,446 (5.5%) | 709 (2.7%) | 4,893 (18.8%) | 3,108 (11.9%) | 5,786 (22.3%) | 3,696 (14.2%) | 4,050 (15.6%) | 2,290 (8.8%) |
|  | Mean | 78.5 | 75.9 | 89.5 | 75.7 | 88.8 | 74.5 | 86.3 | 70.5 | 80.7 |
|  | SD | 16.0 | 16.3 | 17.4 | 15.5 | 16.2 | 14.2 | 14.7 | 13.1 | 12.2 |
| **Height (m)** |  | 25,978 (100%) | 1,446 (5.5%) | 709 (2.7%) | 4,893 (18.8%) | 3,108 (11.9%) | 5,786 (22.3%) | 3,696 (14.2%) | 4,050 (15.6%) | 2,290 (8.8%) |
|  | Mean | 1.67 | 1.64 | 1.77 | 1.64 | 1.76 | 1.62 | 1.75 | 1.61 | 1.73 |
|  | SD | 0.09 | 0.06 | 0.07 | 0.06 | 0.07 | 0.06 | 0.07 | 0.06 | 0.07 |
| **BMI (kg/m²)** |  | 25,978 (100%) | 1,446 (5.5%) | 709 (2.7%) | 4,893 (18.8%) | 3,108 (11.9%) | 5,786 (22.3%) | 3,696 (14.2%) | 4,050 (15.6%) | 2,290 (8.8%) |
|  | Mean | 27.9 | 27.9 | 28.3 | 28.1 | 28.6 | 28.2 | 28.1 | 27.1 | 26.9 |
|  | SD | 5.10 | 5.84 | 5.10 | 5.57 | 4.89 | 5.27 | 4.64 | 4.93 | 3.88 |
| **Years of schooling** | 0-6 years | 3,320 (12.8%) | 113 (7.8%) | 63 (8.9%) | 444 (9.1%) | 214 (6.9%) | 717 (12.4%) | 367 (9.9%) | 955 (23.6%) | 447 (19.5%) |
|  | 7-12 years | 14,235 (54.8%) | 727 (50.3%) | 344 (48.5%) | 2,614 (53.4%) | 1,826 (58.8%) | 3,322 (57.4%) | 2,015 (54.5%) | 2,180 (53.8%) | 1,207 (52.7%) |
|  | 13-18 years | 7,594 (29.2%) | 534 (36.9%) | 262 (37.0%) | 1,664 (34.0%) | 954 (30.7%) | 1,615 (27.9%) | 1,169 (31.6%) | 850 (21.0%) | 546 (23.8%) |
|  | 19+ years | 829 (3.2%) | 72 (5.0%) | 40 (5.6%) | 171 (3.5%) | 114 (3.7%) | 132 (2.3%) | 145 (3.9%) | 65 (1.6%) | 90 (3.9%) |
| **Incontinence** |  | 1,126 (4.3%) | 26 (1.8%) | 6 (0.8%) | 119 (2.4%) | 20 (0.6%) | 251 (4.3%) | 80 (2.2%) | 497 (12.3%) | 127 (5.5%) |
| **Handgrip strength** |  | 2155 (8.3%) | 1446 (67.1%) | 709 (32.9%) | 4893 (61.2%) | 3108 (38.8%) | 5786 (61.0%) | 3696 (39.0%) | 4050 (63.9%) | 2290 (36.1%) |
| **Weight loss** |  | 6,097 (23.5%) | 324 (22.4%) | 183 (25.8%) | 1,126 (23.0%) | 673 (21.7%) | 1,325 (22.9%) | 831 (22.5%) | 1,053 (26.0%) | 582 (25.4%) |
| **Multiple morbidities (≥3)** |  | 8,646 (33.3%) | 200 (13.8%) | 121 (17.1%) | 1,201 (24.5%) | 749 (24.1%) | 2,196 (37.9%) | 1,298 (35.1%) | 1,885 (46.5%) | 996 (43.5%) |
| **Depressive symptoms** |  | 11,539 (44.4%) | 720 (49.8%) | 279 (39.4%) | 2,303 (47.1%) | 1,041 (33.5%) | 2,799 (48.4%) | 1,151 (31.1%) | 2,277 (56.2%) | 969 (42.3%) |
| **Self-rated health** | Good | 12,068 (46.5%) | 816 (56.4%) | 405 (57.1%) | 2,624 (53.6%) | 1,613 (51.9%) | 2,683 (46.4%) | 1,785 (48.3%) | 1,314 (32.4%) | 828 (36.2%) |
|  | Regular | 10,385 (40.0%) | 491 (34.0%) | 202 (28.5%) | 1,769 (36.2%) | 1,127 (36.3%) | 2,363 (40.8%) | 1,443 (39.0%) | 1,950 (48.1%) | 1,040 (45.4%) |
|  | Poor | 3,525 (13.6%) | 139 (9.6%) | 102 (14.4%) | 500 (10.2%) | 368 (11.8%) | 740 (12.8%) | 468 (12.7%) | 786 (19.4%) | 422 (18.4%) |
| **Memory** | Good | 17,523 (67.5%) | 1,145 (79.2%) | 555 (78.3%) | 3,701 (75.6%) | 2,295 (73.8%) | 3,952 (68.3%) | 2,484 (67.2%) | 2,197 (54.2%) | 1,194 (52.1%) |
|  | Regular | 7,194 (27.7%) | 260 (18.0%) | 124 (17.5%) | 1,063 (21.7%) | 712 (22.9%) | 1,603 (27.7%) | 1,038 (28.1%) | 1,500 (37.0%) | 894 (39.0%) |
|  | Poor | 1,261 (4.9%) | 41 (2.8%) | 30 (4.2%) | 129 (2.6%) | 101 (3.3%) | 231 (4.0%) | 174 (4.7%) | 353 (8.7%) | 202 (8.8%) |
| **Vision** | Good | 19,378 (74.6%) | 1,151 (79.6%) | 533 (75.2%) | 3,899 (79.7%) | 2,476 (79.7%) | 4,328 (74.8%) | 2,897 (78.4%) | 2,544 (62.8%) | 1,550 (67.7%) |
|  | Regular | 4,960 (19.1%) | 201 (13.9%) | 128 (18.1%) | 760 (15.5%) | 486 (15.6%) | 1,122 (19.4%) | 619 (16.7%) | 1,088 (26.9%) | 556 (24.3%) |
|  | Poor | 1,640 (6.3%) | 94 (6.5%) | 48 (6.8%) | 234 (4.8%) | 146 (4.7%) | 336 (5.8%) | 180 (4.9%) | 418 (10.3%) | 184 (8.0%) |
| **Hearing** | Good | 20,268 (78.0%) | 1,306 (90.3%) | 590 (83.2%) | 4,342 (88.7%) | 2,503 (80.5%) | 4,736 (81.8%) | 2,698 (73.0%) | 2,685 (66.3%) | 1,408 (61.5%) |
|  | Regular | 4,742 (18.3%) | 118 (8.2%) | 96 (13.5%) | 493 (10.1%) | 520 (16.7%) | 888 (15.3%) | 853 (23.1%) | 1,085 (26.8%) | 689 (30.1%) |
|  | Poor | 968 (3.7%) | 22 (1.5%) | 23 (3.2%) | 58 (1.2%) | 85 (2.7%) | 162 (2.8%) | 145 (3.9%) | 280 (6.9%) | 193 (8.4%) |
| **BADL limitation (>1)** |  | 3,690 (14.2%) | 125 (8.6%) | 85 (12.0%) | 466 (9.5%) | 366 (11.8%) | 748 (12.9%) | 478 (12.9%) | 931 (23.0%) | 491 (21.4%) |
| **IADL limitation (>1)** |  | 3,590 (13.8%) | 83 (5.7%) | 51 (7.2%) | 318 (6.5%) | 161 (5.2%) | 711 (12.3%) | 339 (9.2%) | 1,375 (33.9%) | 552 (24.1%) |

BADL: basic activities of daily living; BMI: body mass index; IADL: instrumental activities of daily living; kg/m²: kilogram per square meter; SD: standard deviation.
