# Supplementary material for: A decade of difference: cross-continental patterns of functional performance in Brazil and Europe within the WHO ICOPE framework
Source: PLoS One. 2026 Aug 5;21(8):e0353750. doi: 10.1371/journal.pone.0353750 (PMC13440855; doi:10.1371/journal.pone.0353750)
Supplement: Peer review history

# Peer review: Decision Letter 0

**DOI:** 10.1371/journal.pone.0353750.r001

**Authors:** Yogesh Jain

27 May 2026

PONE-D-26-11227

A decade of difference: cross-continental patterns of functional performance in Brazil and Europe within the WHO ICOPE framework

PLOS One

Dear Dr. Oliveira,

Thank you for submitting your manuscript to PLOS ONE. After careful consideration, we feel that it has merit but does not fully meet PLOS ONE’s publication criteria as it currently stands. Therefore, we invite you to submit a revised version of the manuscript that addresses the points raised during the review process.

Please submit your revised manuscript by Jul 11 2026 11:59PM. If you will need more time than this to complete your revisions, please reply to this message or contact the journal office at plosone@plos.org. When you're ready to submit your revision, log on to https://www.editorialmanager.com/pone/ and select the 'Submissions Needing Revision' folder to locate your manuscript file.

Please include the following items when submitting your revised manuscript:

- A letter that responds to each point raised by the academic editor and reviewer(s). You should upload this letter as a separate file labeled 'Response to Reviewers'.
- A marked-up copy of your manuscript that highlights changes made to the original version. You should upload this as a separate file labeled 'Revised Manuscript with Track Changes'.
- An unmarked version of your revised paper without tracked changes. You should upload this as a separate file labeled 'Manuscript'.

If you would like to make changes to your financial disclosure, please include your updated statement in your cover letter. Guidelines for resubmitting your figure files are available below the reviewer comments at the end of this letter.

If applicable, we recommend that you deposit your laboratory protocols in protocols.io to enhance the reproducibility of your results. Protocols.io assigns your protocol its own identifier (DOI) so that it can be cited independently in the future. For instructions see: https://journals.plos.org/plosone/s/submission-guidelines#loc-laboratory-protocols. Additionally, PLOS ONE offers an option for publishing peer-reviewed Lab Protocol articles, which describe protocols hosted on protocols.io. Read more information on sharing protocols at https://plos.org/protocols?utm_medium=editorial-email&utm_source=authorletters&utm_campaign=protocols.

As the corresponding author, your ORCID iD is verified in the submission system and will appear in the published article. PLOS supports the use of ORCID, and we encourage all coauthors to register for an ORCID iD and use it as well. Please encourage your coauthors to verify their ORCID iD within the submission system before final acceptance, as unverified ORCID iDs will not appear in the published article. *Only*  the individual author can complete the verification step; PLOS staff *cannot*  verify ORCID iDs on behalf of authors.

We look forward to receiving your revised manuscript.

Kind regards,

Yogesh Kumar Jain, PhD

Academic Editor

PLOS One

Journal Requirements:

When submitting your revision, we need you to address these additional requirements.

1.Please ensure that your manuscript meets PLOS ONE's style requirements, including those for file naming. The PLOS ONE style templates can be found at

https://journals.plos.org/plosone/s/file?id=wjVg/PLOSOne_formatting_sample_main_body.pdf and

https://journals.plos.org/plosone/s/file?id=ba62/PLOSOne_formatting_sample_title_authors_affiliations.pdf

2. Thank you for uploading your study's underlying data set. Unfortunately, the repository you have noted in your Data Availability statement does not qualify as an acceptable data repository according to PLOS's standards.

At this time, please upload the minimal data set necessary to replicate your study's findings to a stable, public repository (such as figshare or Dryad) and provide us with the relevant URLs, DOIs, or accession numbers that may be used to access these data. For a list of recommended repositories and additional information on PLOS standards for data deposition, please see https://journals.plos.org/plosone/s/recommended-repositories.

3. Your ethics statement should only appear in the Methods section of your manuscript. If your ethics statement is written in any section besides the Methods, please move it to the Methods section and delete it from any other section. Please ensure that your ethics statement is included in your manuscript, as the ethics statement entered into the online submission form will not be published alongside your manuscript.

4. If the reviewer comments include a recommendation to cite specific previously published works, please review and evaluate these publications to determine whether they are relevant and should be cited. There is no requirement to cite these works unless the editor has indicated otherwise.

5. Please review your reference list to ensure that it is complete and correct. If you have cited papers that have been retracted, please include the rationale for doing so in the manuscript text, or remove these references and replace them with relevant current references. Any changes to the reference list should be mentioned in the rebuttal letter that accompanies your revised manuscript. If you need to cite a retracted article, indicate the article’s retracted status in the References list and also include a citation and full reference for the retraction notice.

Additional Editor Comments:

The manuscript has received an overall positive feedback by the reviewers. However, I would recommend some minor revisions based on the suggestions, especially from reviewer #2. This would greatly improve the clarity of the messages conveyed.

[Note: HTML markup is below. Please do not edit.]

Reviewer's Responses to Questions

**Comments to the Author**

1. Is the manuscript technically sound, and do the data support the conclusions?

The manuscript must describe a technically sound piece of scientific research with data that supports the conclusions. Experiments must have been conducted rigorously, with appropriate controls, replication, and sample sizes. The conclusions must be drawn appropriately based on the data presented.

Reviewer #1: Yes

Reviewer #2: Yes

**********

2. Has the statistical analysis been performed appropriately and rigorously?

Reviewer #1: Yes

Reviewer #2: Yes

**********

3. Have the authors made all data underlying the findings in their manuscript fully available?

The PLOS Data policy requires authors to make all data underlying the findings described in their manuscript fully available without restriction, with rare exception (please refer to the Data Availability Statement in the manuscript PDF file). The data should be provided as part of the manuscript or its supporting information, or deposited to a public repository. For example, in addition to summary statistics, the data points behind means, medians and variance measures should be available. If there are restrictions on publicly sharing data—e.g. participant privacy or use of data from a third party—those must be specified.

Reviewer #1: Yes

Reviewer #2: Yes

**********

4. Is the manuscript presented in an intelligible fashion and written in standard English?

PLOS ONE does not copyedit accepted manuscripts, so the language in submitted articles must be clear, correct, and unambiguous. Any typographical or grammatical errors should be corrected at revision, so please note any specific errors here.

Reviewer #1: Yes

Reviewer #2: Yes

**********

5. Review Comments to the Author

Please use the space provided to explain your answers to the questions above. You may also include additional comments for the author, including concerns about dual publication, research ethics, or publication ethics. (Please upload your review as an attachment if it exceeds 20,000 characters)

Reviewer #1: The manuscript presents clear and coherent objectives, and the methodology is appropriate to address the research question, with a sufficient sample size and the use of valid and reliable instruments. The results are clearly presented, and the conclusions are supported by the data. The statistical approach appears adequate for the aims of the study. In addition, the study uses data derived from two public datasets/projects, and therefore the underlying data are publicly available, supporting transparency and reproducibility. The manuscript is well written, clear, and easy to understand. The objectives, methods, and results are presented in a logical and coherent manner, and the English language is appropriate for scientific publication.

Reviewer #2: I congratulate the authors on the manuscript.

Some issues could be better described: was the self-reported sensory assessment (vision and hearing) based on any literature? If so, they should cite it. Do the authors believe that elderly people know how to identify hearing loss?

Table 2 shows that the average ages of the two groups appear to be quite different. Was a statistical analysis performed on this? Could this have influenced the results?

Of course we have to believe the participants' answers, but my experience when evaluating IADLs and BADLs is that men lie in their responses. Do you have anything to say about that?

Typically, handgrip strength assessment is used to evaluate the vitality domain. Why did the authors use it in the locomotor domain?

The data collection for both ELSI-Brazil (second wave) and SHARE (ninth wave) occurred during the COVID-19 pandemic. Could this have interfered? The ELSI-Brazil study was collected during the worst phase of the pandemic, while SHARE was collected when the pandemic was more controlled in Europe. Could this have interfered? None of this was discussed in the manuscript.

I missed seeing practical and clinical applications for the results. What suggestions do the authors have for improving healthy aging, considering the study's findings?

The authors should state the limitations of the study.

**********

6. PLOS authors have the option to publish the peer review history of their article (what does this mean?). If published, this will include your full peer review and any attached files.

If you choose “no”, your identity will remain anonymous but your review may still be made public.

**Do you want your identity to be public for this peer review?**  For information about this choice, including consent withdrawal, please see our Privacy Policy.

Reviewer #1: **Yes:** José Vinícius Alves Ferreira

Reviewer #2: **Yes:** Marcos Eduardo Scheicher

**********

[NOTE: If reviewer comments were submitted as an attachment file, they will be attached to this email and accessible via the submission site. Please log into your account, locate the manuscript record, and check for the action link "View Attachments". If this link does not appear, there are no attachment files.]

To ensure your figures meet our technical requirements, please review our figure guidelines: https://journals.plos.org/plosone/s/figures

You may also use PLOS’s free figure tool, NAAS, to help you prepare publication quality figures: https://journals.plos.org/plosone/s/figures#loc-tools-for-figure-preparation.

NAAS will assess whether your figures meet our technical requirements by comparing each figure against our figure specifications.

# Author comment: Author response to Decision Letter 1

**DOI:** 10.1371/journal.pone.0353750.r002

2 Jun 2026

Additional Editor Comments:

The manuscript has received an overall positive feedback by the reviewers. However, I would recommend some minor revisions based on the suggestions, especially from reviewer #2. This would greatly improve the clarity of the messages conveyed.

R: Thank you for your positive feedback. The comments of both reviewers have been addressed accordingly.

________________________________________

Review Comments to the Author

Reviewer #1: The manuscript presents clear and coherent objectives, and the methodology is appropriate to address the research question, with a sufficient sample size and the use of valid and reliable instruments. The results are clearly presented, and the conclusions are supported by the data. The statistical approach appears adequate for the aims of the study. In addition, the study uses data derived from two public datasets/projects, and therefore the underlying data are publicly available, supporting transparency and reproducibility. The manuscript is well written, clear, and easy to understand. The objectives, methods, and results are presented in a logical and coherent manner, and the English language is appropriate for scientific publication.

R: We are pleased that the reviewer found the manuscript to be well written, logically structured, and easy to follow. We have carefully revised the manuscript to ensure that this level of clarity is maintained throughout and have made minor editorial improvements where appropriate. Thank you for your valuable feedback and support.

Reviewer #2: I congratulate the authors on the manuscript.

Some issues could be better described: was the self-reported sensory assessment (vision and hearing) based on any literature? If so, they should cite it.

R: Thank you for this comment. Actually, the self-reported sensory assessment (vision and hearing) used both in ELSI-Brasil and SHARE are subjectivelly collected using a 5-point Likert-type scale. These assessments in both datasets have common similarities such as the inclusion of correction devices (glasses/hearing aids) in the evaluation, and the use of ordinal scales reflecting perceived ability. Overall, they capture functional sensory capacity rather than clinical diagnosis, in accordance with the WHO ICOPE framework. The references have been cited accordingly (line 150).

Do the authors believe that elderly people know how to identify hearing loss?

R: Thank you for this important and thought-provoking comment. While self-reported hearing measures rely on an individual’s perception, evidence suggests that older adults are able to reasonably identify functional hearing difficulties, particularly when these limitations affect daily communication. Given that both ELSI-Brasil and SHARE use harmonized self-reported questions (including the use of hearing aids), these measures remain appropriate and widely accepted for large population-based studies, particularly in cross-national comparisons where objective testing is not feasible. We have added a brief clarification in the manuscript acknowledging this limitation (lines 350-353).

Table 2 shows that the average ages of the two groups appear to be quite different. Was a statistical analysis performed on this? Could this have influenced the results?

R: We thank the reviewer for this important observation. We performed Welch's independent samples t-test to formally compare the mean age between the two cohorts, which confirms the statistically significant difference (ELSI-Brasil: 64.9 ±

9.0 years; SHARE: 72.6 ± 9.4 years; t = -54.19, df = 6924.03, p < 0.001).

We fully agree that age is a critical factor that could potentially influence the results, given its strong association with funcional decline. Therefore, age was pre-defined and included as a sociodemographic covariate in all multivariable logistic regression

models. The reported associations for all outcomes are thus statistically adjusted for age, sex, and education, which substantially minimizes the risk of confounding by the age difference between the two samples. We have now added a clarifying statement in the Methods section explaining the inclusion of age as a covariate to control for potential confounding (lines 190-192), and we have inserted the p-value in Table 2 (footnote, line 203).

Of course we have to believe the participants' answers, but my experience when evaluating IADLs and BADLs is that men lie in their responses. Do you have anything to say about that?

R: Thank you for raising this important point. We agree that self-reported functional assessments may be influenced by gender-related social expectations and reporting behavior, particularly among men, who may underreport dependency due to norms associated with masculinity and autonomy. Accordingly, we acknowledge that self-reported measures of BADLs and IADLs may be subject to reporting bias, including potential differences by sex. However, self-reported functional assessments remain the standard approach in large population-based studies such as ELSI-Brasil and SHARE, given their feasibility and their ability to capture perceived functional capacity in daily life, thereby ensuring consistency and comparability across populations. We have acknowledged this as a potential limitation in the manuscript and highlighted the need for future studies combining self-reported and objective functional measures (lines 353–358).

Typically, handgrip strength assessment is used to evaluate the vitality domain. Why did the authors use it in the locomotor domain?

R: Thank you for this insightful comment. In our study, handgrip strength was included within the mobility domain because it reflects neuromuscular performance and functional reserve associated with locomotion and disability risk. Although measured in the upper limb, handgrip strength is widely recognized as a proxy for global muscle strength, and has been strongly associated with mobility outcomes such as gait speed, chair rise performance, and risk of physical disability and frailty (Abdalla et al., 2023, doi: 10.1016/j.archger.2022.104869; McGrath et al., 2022, doi: 10.1016/j.archger.2022.104716). This approach also ensured consistency across both datasets, where comparable locomotor performance measures were limited.

The data collection for both ELSI-Brazil (second wave) and SHARE (ninth wave) occurred during the COVID-19 pandemic. Could this have interfered? The ELSI-Brazil study was collected during the worst phase of the pandemic, while SHARE was collected when the pandemic was more controlled in Europe. Could this have interfered? None of this was discussed in the manuscript.

R: We thank the reviewer for raising this highly relevant point. We agree that ELSI-Brasil Wave 2 (2019-2021) was collected during a more critical phase of the COVID-19 pandemic in Brazil, whereas SHARE Wave 9 (2021-2022) occurred during a comparatively more controlled phase in Europe. Therefore, the pandemic context may have influenced functional performance, mental health, mobility, and self-perception outcomes in both cohorts. However, pandemic-related factors are unlikely to fully account for the observed patterns, especially given the consistency of key associations across domains and populations. We added this limitation into the Discussion (lines 358-364) and thank the reviewer for helping us improve the contextual interpretation of our findings.

I missed seeing practical and clinical applications for the results. What suggestions do the authors have for improving healthy aging, considering the study's findings?

R: We thank the reviewer for prompting us to strengthen the clinical relevance of our work. We have now incorporated the clinical implications of our findings alongside the policy implications in the Discussion section (lines 335-345).

The authors should state the limitations of the study.

R: The “limitations” paragraph has been updated in accordance with the reviewer’s comments (lines 346-364).

We sincerely appreciate the reviewer’s careful evaluation and believe the manuscript has been substantially strengthened following these recommendations.

Yours sincerely,

The authors

Attachment Submitted filename: Response to Reviewers.docx

# Peer review: Decision Letter 1

**DOI:** 10.1371/journal.pone.0353750.r003

**Authors:** Yogesh Jain, Yogesh Jain

29 Jun 2026

<p>A decade of difference: cross-continental patterns of functional performance in Brazil and Europe within the WHO ICOPE framework

PONE-D-26-11227R1

Dear Dr. Oliveira,

We’re pleased to inform you that your manuscript has been judged scientifically suitable for publication and will be formally accepted for publication once it meets all outstanding technical requirements.

Within one week, you’ll receive an e-mail detailing the required amendments. When these have been addressed, you’ll receive a formal acceptance letter and your manuscript will be scheduled for publication.

An invoice will be generated when your article is formally accepted. Please note, if your institution has a publishing partnership with PLOS and your article meets the relevant criteria, all or part of your publication costs will be covered. Please make sure your user information is up-to-date by logging into Editorial Manager at Editorial Manager® and clicking the ‘Update My Information' link at the top of the page. For questions related to billing, please contact billing support.

If your institution or institutions have a press office, please notify them about your upcoming paper to help maximize its impact. If they’ll be preparing press materials, please inform our press team as soon as possible -- no later than 48 hours after receiving the formal acceptance. Your manuscript will remain under strict press embargo until 2 pm Eastern Time on the date of publication. For more information, please contact onepress@plos.org.

Kind regards,

Yogesh Kumar Jain, PhD

Academic Editor

PLOS One

Additional Editor Comments (optional):

Reviewers' comments:

Reviewer's Responses to Questions

**Comments to the Author**

1. If the authors have adequately addressed your comments raised in a previous round of review and you feel that this manuscript is now acceptable for publication, you may indicate that here to bypass the “Comments to the Author” section, enter your conflict of interest statement in the “Confidential to Editor” section, and submit your "Accept" recommendation.

Reviewer #1: All comments have been addressed

Reviewer #2: All comments have been addressed

**********

2. Is the manuscript technically sound, and do the data support the conclusions?

The manuscript must describe a technically sound piece of scientific research with data that supports the conclusions. Experiments must have been conducted rigorously, with appropriate controls, replication, and sample sizes. The conclusions must be drawn appropriately based on the data presented.

Reviewer #1: Yes

Reviewer #2: Yes

**********

3. Has the statistical analysis been performed appropriately and rigorously?

Reviewer #1: Yes

Reviewer #2: Yes

**********

4. Have the authors made all data underlying the findings in their manuscript fully available?

The PLOS Data policy requires authors to make all data underlying the findings described in their manuscript fully available without restriction, with rare exception (please refer to the Data Availability Statement in the manuscript PDF file). The data should be provided as part of the manuscript or its supporting information, or deposited to a public repository. For example, in addition to summary statistics, the data points behind means, medians and variance measures should be available. If there are restrictions on publicly sharing data—e.g. participant privacy or use of data from a third party—those must be specified.

Reviewer #1: Yes

Reviewer #2: Yes

**********

5. Is the manuscript presented in an intelligible fashion and written in standard English?

PLOS ONE does not copyedit accepted manuscripts, so the language in submitted articles must be clear, correct, and unambiguous. Any typographical or grammatical errors should be corrected at revision, so please note any specific errors here.

Reviewer #1: Yes

Reviewer #2: Yes

**********

6. Review Comments to the Author

Please use the space provided to explain your answers to the questions above. You may also include additional comments for the author, including concerns about dual publication, research ethics, or publication ethics. (Please upload your review as an attachment if it exceeds 20,000 characters)

Reviewer #1: I appreciate the authors' efforts in addressing the comments raised during the previous review round. The revised manuscript has been improved, and the authors have adequately responded to the main concerns.

I maintain my opinion that this manuscript meets the scientific standards of the journal and is suitable for publication. The study addresses a relevant topic, the methodology is appropriate, and the findings contribute to the current literature. While minor limitations remain, they do not compromise the overall quality or validity of the work.

Therefore, I support the publication of the manuscript in its current form.

Reviewer #2: I congratulate the authors again on the article. It is an important topic, still little explored and, perhaps, a possibility for a paradigm shift in healthcare practices regarding the elderly. The changes were accepted and included in the text.

**********

7. PLOS authors have the option to publish the peer review history of their article (what does this mean?). If published, this will include your full peer review and any attached files.

If you choose “no”, your identity will remain anonymous but your review may still be made public.

**Do you want your identity to be public for this peer review?**  For information about this choice, including consent withdrawal, please see our Privacy Policy.

Reviewer #1: **Yes:** José Vinícius Alves Ferreira

Reviewer #2: **Yes:** Marcos Eduardo Scheicher

**********

# Editor report: Acceptance letter

**DOI:** 10.1371/journal.pone.0353750.r004

**Authors:** Yogesh Jain, Yogesh Jain

PONE-D-26-11227R1

PLOS One

Dear Dr. Oliveira,

I'm pleased to inform you that your manuscript has been deemed suitable for publication in PLOS One. Congratulations! Your manuscript is now being handed over to our production team.

At this stage, our production department will prepare your paper for publication. This includes ensuring the following:

* All references, tables, and figures are properly cited

* All relevant supporting information is included in the manuscript submission,

* There are no issues that prevent the paper from being properly typeset

You will receive further instructions from the production team, including instructions on how to review your proof when it is ready. Please keep in mind that we are working through a large volume of accepted articles, so please give us a few days to review your paper and let you know the next and final steps.

Lastly, if your institution or institutions have a press office, please let them know about your upcoming paper now to help maximize its impact. If they'll be preparing press materials, please inform our press team within the next 48 hours. Your manuscript will remain under strict press embargo until 2 pm Eastern Time on the date of publication. For more information, please contact onepress@plos.org.

You will receive an invoice from PLOS for your publication fee after your manuscript has reached the completed accept phase. If you receive an email requesting payment before acceptance or for any other service, this may be a phishing scheme. Learn how to identify phishing emails and protect your accounts at https://explore.plos.org/phishing.

If we can help with anything else, please email us at customercare@plos.org.

Thank you for submitting your work to PLOS ONE and supporting open access.

Kind regards,

PLOS ONE Editorial Office Staff

on behalf of

Dr. Yogesh Kumar Jain

Academic Editor

PLOS One
